# Molecular epidemiology of mosquito-borne viruses at the China–Myanmar border: discovery of a potential epidemic focus of Japanese encephalitis

**DOI:** 10.1186/s40249-021-00838-z

**Published:** 2021-04-26

**Authors:** Yuan Fang, Xi-Shang Li, Wei Zhang, Jing-Bo Xue, Jia-Zhi Wang, Shou-Qin Yin, Sheng-Guo Li, Xin-He Li, Yi Zhang

**Affiliations:** 1 National Institute of Parasitic Diseases, Chinese Center for Disease Control and Prevention (Chinese Center for Tropical Diseases Research); NHC Key Laboratory of Parasite and Vector Biology; WHO Collaborating Centre for Tropical Diseases; National Center for International Research on Tropical Diseases, Shanghai, China; 2 School of Global Health, Chinese Center for Tropical Diseases Research, Shanghai Jiao Tong University School of Medicine, Shanghai, China; 3 Tengchong County Center for Disease Control and Prevention, Tengchong, Yunnan China; 4 Zichuan District Center for Disease Control and Prevention, Shandong Zibo, China

**Keywords:** *Culex tritaeniorhynchus*, Insect-specific flavivirus, Mosquito-borne diseases, Tengchong, China, SA14-14-2

## Abstract

**Background:**

Mosquito-based arbovirus surveillance can serve as an early warning in evaluating the status of mosquito-borne virus prevalence and thus prevent local outbreaks. Although Tengchong County in Yunnan Province—which borders Myanmar—is abundant and diverse in mosquitoes, very few mosquito-based arbovirus investigations have been conducted in the recent decade. Herein, this study aims to evaluate the presence and the diffusion of mosquito-borne pathogens, currently prevalent in this region.

**Methods:**

We collected 9486 mosquitoes, representing eight species, with *Culex tritaeniorhynchus* and *Anopheles sinensis* as the dominant species, during high mosquito activity seasons (July–October) in Tengchong, in 2018. Samples collected from 342 pools were tested using reverse-transcription PCR to determine the species, distribution, and infection rates of virus and parasite, and further analyze their genotypes, phylogenetic relationships, infection rate, and potential pathogenicity.

**Results:**

Fifteen Japanese encephalitis virus (JEV) strains from *Cx. tritaeniorhynchus* pools were detected. Seven strains of insect-specific flaviviruses (ISFVs), including two Aedes flavivirus (AeFV) and Yunnan Culex flavivirus strains each, one Culex theileri flavivirus, Yamadai flavivirus (YDFV) and Anopheles-associated flavivirus (AAFV) strains each were detected in *Aedes albopictus*, *Cx. tritaeniorhynchus*, *Cx. vagans*, *Cx. pseudovihnui*, and *An. sinensis* pools, respectively. The whole-genome was successfully amplified in one strain of JEV and AeFV each. Phylogenetic analysis using the *E* gene placed all the newly detected JEV strains into the GI-b genotype. They showed highly nucleotide identities, and were most closely related to the strain detected in Tengchong in 2010. The comparison of the E protein of JEV strains and vaccine-derived strain, showed six amino residue differences. The bias-corrected maximum likelihood estimation values (and 95% confidence interval) for JEV in *Cx. tritaeniorhynchus* collected in Tengchong in 2018 were 2.4 (1.4–3.9).

**Conclusions:**

A potential Japanese encephalitis epidemic focus with the abundance of host mosquitoes and high JEV infection rate was observed in Tengchong. In addition, at least five species of ISFVs co-circulate in this area. This study highlights the importance of widespread and sustained mosquito-based arbovirus surveillance in local areas to prevent the transmission of JEV, and other emerging/re-emerging mosquito-borne pathogens.

**Graphic Abstract:**

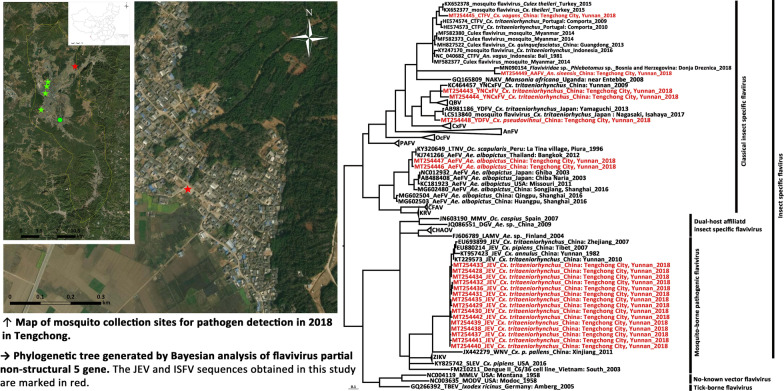

**Supplementary Information:**

The online version contains supplementary material available at 10.1186/s40249-021-00838-z.

## Background

The prevalence of mosquito-borne viruses is rising steeply with increasing frequency of international trade and travel. Because of the existence of vectors, imported mosquito-borne diseases may cause local infections and even outbreaks in areas with no previous record of a specific mosquito-borne pathogen detection.

The majority of China is located in warm-tropical, subtropical, and tropical zones, which are suitable for the breeding of mosquitoes. Mosquitoes which transmit clinically relevant viruses in China include *Aedes albopictus*, *Culex tritaeniorhynchus*, and *Anopheles sinensis* as the dominant species in several areas, mainly transmitting dengue virus (DENV), Japanese encephalitis virus (JEV), and *Plasmodium* spp., respectively. Dengue outbreaks occurred in Yunnan (2013, [1]), Guangdong (2014, [2]), Fujian (2016, [3]), and Zhejiang (2017, [4]) provinces throughout the last decades. Despite the implementation and expansion of Japanese encephalitis (JE) vaccination program for children in China since the 1970s, and 2008, respectively, there were occasional incidents of JE recurrence and outbreaks in Shanxi (2006, [5]), Hubei (2009–2010, [6]), Shandong (2013, [7]), and Gansu (2018, [8]) provinces. The increase in adult JE cases is a characteristic of JE outbreaks in recent years. China has applied to the World Health Organization for the certification of malaria eradication in December 2020 [9], and thus monitoring local malaria cases and preventing secondary cases would be of focus in the future. China was a traditionally Chikungunya-free country, until 2010, when the first chikungunya outbreak was reported in Guangdong that resulted in 173 clinical cases [10]. The Zika virus (ZIKV) was prevalent in South America in 2016 [11], in the same year, it was detected in mosquitoes in Guizhou [12, 13] and Yunnan [14], indicating the circulation of ZIKV in China before it was widespread.

Due to the geological position and complex natural climate, the mosquitoes in the Yunnan Province are abundant and diversified [15, 16]. Clinically relevant mosquitoes, *Cx. tritaeniorhynchus* and *An. sinensis* are active at night and are the dominant species among domestic animals and humans; while, the DENV vector, *Ae. albopictus* is active during the day especially in bamboo and wood groves [17]. Mosquito-borne pathogens, including *Plasmodium* spp., JEV, DENV, ZIKV, Chikungunya virus, Sindbis virus, Banna virus, and several other viruses, have been detected in Yunnan [18–23], which borders with Myanmar, Laos, and Vietnam. Mosquito-borne disease prevention and control in Yunnan is even more complicated and severe for the constant influx of imported cases from neighboring countries [24–26]. The DENV outbreak in Yunnan Province in 2013 was suspected to be induced by the dissemination of previously sporadically reported imported cases of DENV in bordering areas of Yunnan [27]. Tengchong County, adjacent to Myanmar, was once a malaria-unstable region due to the frequent migration of workers from Myanmar [28, 29]. Since 2013, no local cases of malaria have been reported in Tengchong [28, 30]. Moreover, owing to the timely verification of imported malaria cases and prompt investigation on identified malaria foci and further disposal within 7 days after index case reporting, the imported ones have declined since then [29, 31]. Besides parasites, JEV from *Cx. tritaeniorhynchus*, *An. sinensis*, *Armigeres subalbatus*, and Getah virus from *Cx. pseudovishnui* have been isolated from field-collected mosquitoes in 2007 [32, 33]. Tengchong was the second highest county for imported infectious diseases among 25 bordering counties of the Yunnan Province from 2013 to 2017. Dengue fever and malaria were the primary imported diseases in the adjacent areas of Yunnan Province, with an occupation of 62.1% and 36.6%, respectively [31]. Thus, with the wide distribution and abundance of *Cx. tritaeniorhynchus*, *An. sinensis*, and several other clinically relevant mosquitoes in Tengchong [34], it is necessary to document the species diversity, distribution, intensity, genotype variation, and pathogenicity of circulating pathogens in mosquitoes for the development and implementation of disease prevention and control strategies. However, to our knowledge, only a few surveys of arbovirus and parasites in mosquitoes have been reported in Tengchong in the last decade. Therefore, in this study, we used mosquito samples from routine surveillance in Tengchong in 2018 to determine the diversity, geographic distribution and infection rates of *Plasmodium* spp., alphaviruses, flaviviruses, and orthobunyaviruses in mosquitoes, and further analyze their genotypes, phylogenetic relationships, sources of importation, and potential pathogenicity.

## Methods

### Survey site

Tengchong lies in the west of the Yunnan Province, with a 148 km border to Myanmar. It is located at a longitude of 98°05′–98°45′ degrees east, and a latitude of 24°38′–25°52′ degrees north. The altitude is between 930 and 3780 m, decreasing from northwest to southeast. In Tengchong, which is situated in the subtropical monsoon climate, the annual average temperature is 14.9 °C (−4.2–30.5 °C) and the rainfall averages 1531 mm a year, with a mean relative humidity of 77%.

### Sampling

A surveillance program for detecting the distribution and diversity of vector pathogens in field-caught mosquitoes was conducted in Tengchong from July to October 2018. Both ultraviolet light traps (Wuhan Lucky Star Environmental Protection Technology Co., Ltd., Wuhan, China) and labor hour methods were applied to catch adult mosquitoes in five sentinel sites, twice a month, ten days apart. The sentinel sites covered environments of several ecological areas, including the residential area, school, hospital, green space, and livestock farm. The light traps were hung one hour before sunset (7:00 PM) until one hour after sunrise (7:30 AM) to collect the mosquitoes overnight. The labor hour method [35] was used to catch adult mosquitoes in indoor habitats using a mouth aspirator for 15 min, 1 h following sunset.

The survey on the immature growth stage of *Aedes* mosquitoes was conducted once a month in a fixed community, as a sentinel site. Water-holding containers, including tires, rainwater drums, discarded cans, house plants, and other artificial containers closely associated with human dwellings and outdoor environment, were examined for the presence of aedine larvae and pupae. The Breteau index (BI) was calculated by the number of positive containers per 100 houses inspected [36]. The aedine larvae and pupae were brought to the laboratory and reared until the emergence of adults.

**Fig. 1:**
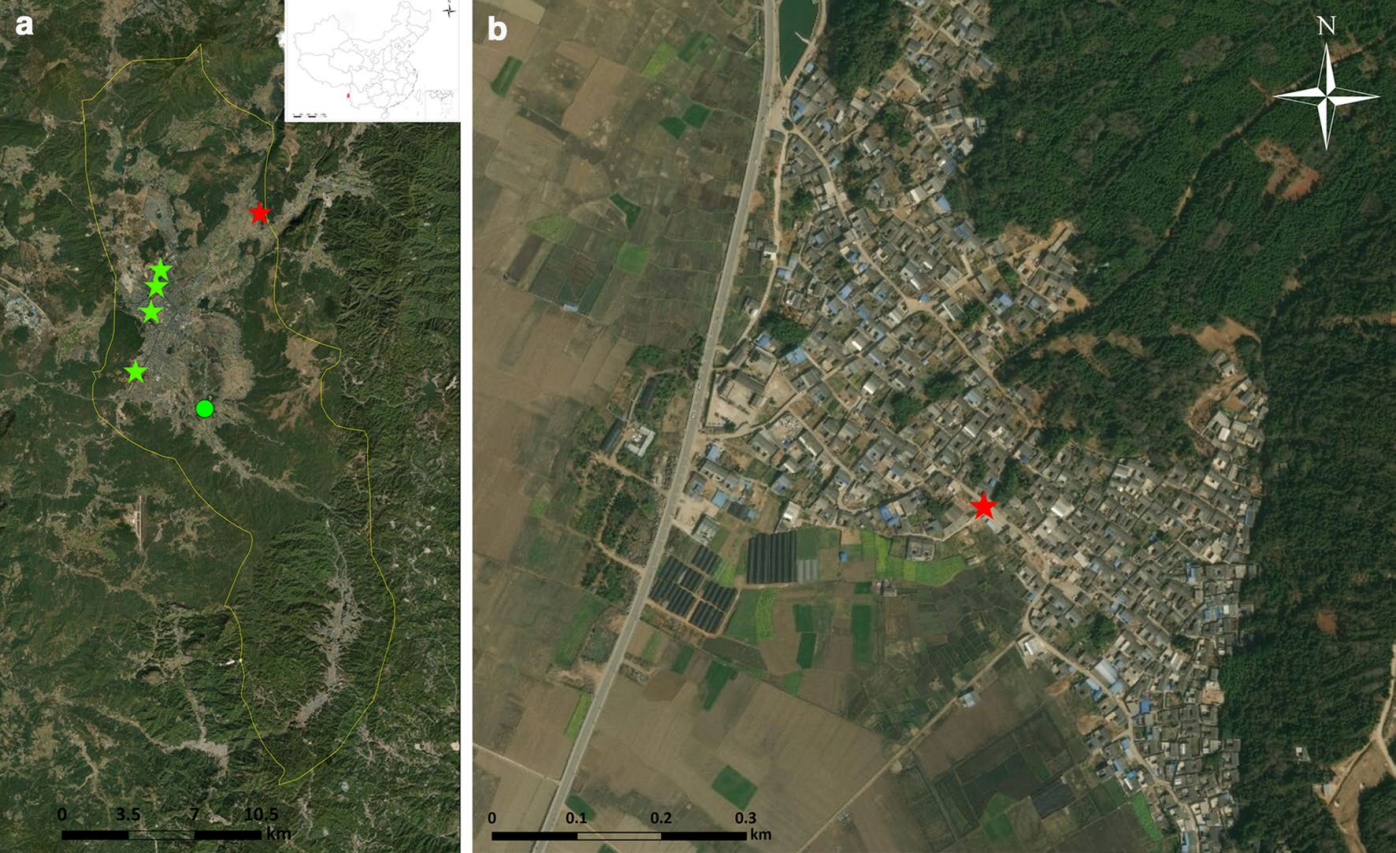
Map of mosquito collection sites for pathogen detection in 2018 in Tengchong. **a** Mosquito collection sites, where the light trap method, labor hour method (represented with stars), and the sentinel site for Breteau index monitoring (represented with a circle) were used. The red-filled star represents the site of Japanese encephalitis virus (JEV) detection. **b** Aerial view of Fuyu village with the settlement and sampling site. The location of the cattle farm is marked with a red-filled star

All the mosquito samples were identified using morphological characteristics according to the national and provincial key [15, 37] under ice bath conditions, and pooled by species, sex, collection date, method and location. Each pool consisted of 1–50 female individuals and was stored at − 80 °C until further pathogen detection. High resolution satellite image from Gaofen-1 satellite at a resolution of 16 m, was obtained for Tengchong from China Center for Resources Satellite Data and Application (www.cresda.com, Accessed 13 March 2021). Collection sites of each method are shown in Fig. 1, and a map was generated using ArcGIS 10.1 ArcMap software (ESRI, Redlands, CA, USA).

### Nucleic acid extraction and polymerase chain reaction (PCR)

RNA was extracted from all pools of mosquitoes using the MagNA Pure 96 System (Roche, Basel, Switzerland) as previously described [38], yielding a final product of 50 μl/pool. First-strand cDNA was synthesized by reverse-transcription (RT) using a Takara PrimeScript RT Reagent Kit with gDNA Eraser (Takara Bio, Shiga, Japan). The mosquito “house-keeping” gene, *18S* rRNA gene was amplified using the RT products with primer set 18S417 and 18S920c [39] to assess the integrity of RNA. Nested multiplex PCR was carried out to detect the presence of four species of *Plasmodium*, including *P. falciparum*, *P. vivax*, *P. malariae*, and *P. ovale*, as described by Li et al. [40]. Flavivirus was amplified using the primer pair PF1S and PF2R-bis [41], and the universal flavivirus primer set cFD2 and MAMD, cFD2 and FS778 by hemi-nested PCR [42], targeting the partial *NS5* gene. Primer sets JEV-Ef/JEV-Er [43], and prMF/prMR [44] were used to amplify the *E* and *prM* genes for further genotype identification. Alphaviruses and orthobunyaviruses in mosquito samples were amplified using primer sets α6533f/α6999c [45] and BCS82C/BCS332V [46], respectively. PCR products were visualized on 1 or 2% (depending on the length of the amplification fragments) agarose gels with Goldview in 0.5 × Tris-acetate-EDTA buffer. Positive products were purified, cloned and sequenced by Sanger sequencing.

### Whole-genome sequencing

For further analyses on the molecular characteristics of the virus, the Primer Premier 5.0 (Premier Biosoft International, Palo Alto, CA, USA) was applied to design primers to amplify the complete genome of JEV and Aedes flavivirus (AeFV). The sequencing of JEV whole-genome was performed using the genomic sequence of the local DH10M978 isolate (GenBank Access No. KT229573) as reference. For sequencing the full-length AeFV, primers were designed using the Thailand strain Bangkok (KJ741266). The resulting PCR products were sent for Sanger sequencing and used to design subsequent primers.

### Phylogenetic analysis

Sequences of PCR products were compared with those deposited in the GenBank database using the BLAST program [47]. Multiple sequence alignments were generated with fragments of relevant flavivirus *NS5* (~ 260 bp), JEV *prM* (~ 580 bp), and JEV *E* (~ 1500 bp) genes, retrieved from GenBank, and sequences obtained in this study, using ClustalW2 [48] with default settings, which were manually adjusted if necessary. Neighbor-joining (NJ) trees were established following Kimura’s two-parameter (K2P) distance model [49] with 1000 bootstrap replicates using MEGA v7.0 software [50]. Based on the Akaike information criterion, the best-fit model for the alignment was determined using Modeltest 3.7 [51], in cooperation with PAUP* v.4.0b10 [52]. Consequently, calculation of the maximum likelihood (ML) and Bayesian likelihood trees was completed under the GTR + I + G model for *NS5* and *E* genes, whereas the TrN + G model was used for the *prM* gene. The ML tree was constructed using MEGA v.7.0 software [50], with 1000 bootstrap replicates. The Bayesian tree was constructed using MrBayes v.3.2.1 [53], run for 10 million generations, with the first 25% of generations discarded as burn-in. The trees were unrooted to provide the least biased topology and visualized using Figtree v.1.4.2 (http://tree.bio.ed.ac.uk/software/figtree/).

### Comparison of virus deduced amino acid sequences

The *E* gene of JEVs sequenced in this study were translated into amino acid sequences and aligned with other representative (homologous) sequences retrieved from GenBank using MEGA v7.0. Amino acid substitutions unique to the newly sequenced strains, and those different from SA14-14-2 vaccine sequence were observed. The attenuated live vaccine SA14-14-2 strain was derived from JEV GIII [54]. In addition, the whole-genome of JEV was translated into amino acid sequences and compared with the closest sequence and the SA14-14-2 strain.

### Infection rate calculation

The size of the pools of collected mosquitoes varied considerably; therefore, infection rates were calculated using bias-corrected maximum likelihood estimation (MLE) and minimum infection rate (MIR) using the Excel add-in PooledInfRate v.4 statistical software package [55]. The rates are expressed as the number of infected mosquitoes per 1000 collected mosquitoes.

## Results

### Mosquito diversity and virus detection from samples

#### Mosquito diversity and density

A total of 9486 mosquitoes, representing eight species were collected in Tengchong during mosquito activity season in 2018, and all the samples in 354 pools were tested for the presence of mosquito-borne pathogens. Among them, *Cx. tritaeniorhynchus* was the dominant species (6490/149 pools), accounted for 68.41%, followed by *An. sinensis* (29.25%, 2775/145 pools), *Cx. pipiens quinquefasciatus* (1.22%, 116/42 pools), *Cx. bitaeniorhynchus* (0.33%, 3/3 pools), *Cx. pseudovihnui* (0.52%, 50/2 pools), *Cx. vagans* (0.02%, 2/1 pool), *Ae. albopictus* (0.45%, 43/7 pools), and *Ar. subalbatus* (0.07%, 7/5 pools). The BI was 0.60, 0.85, 0.63, and 0.24 in July, August, September, and October in Tengchong in 2018, respectively.

#### Molecular identification of mosquito-borne viruses

**Fig. 2:**
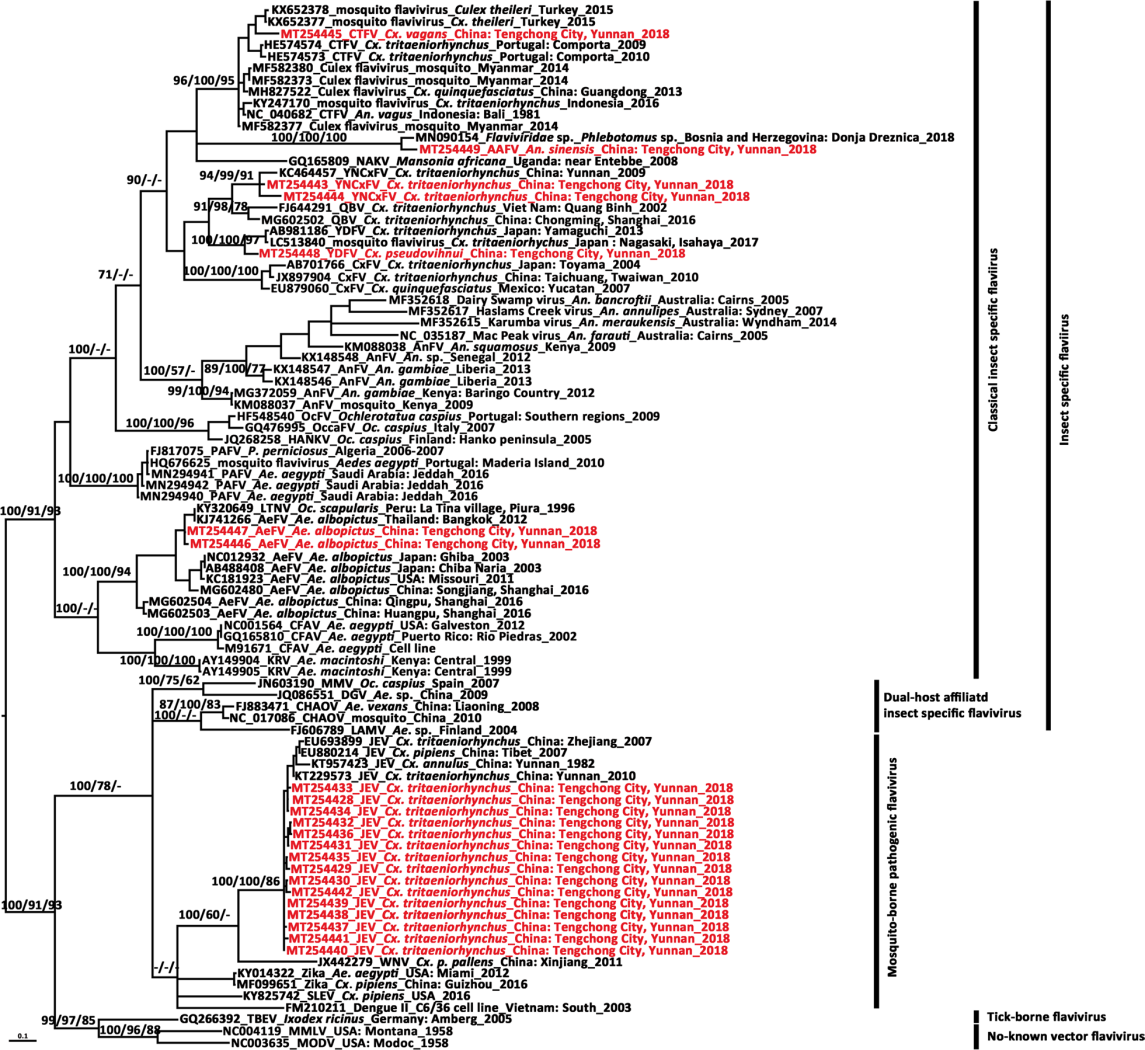
Phylogenetic tree generated by Bayesian analysis of flavivirus partial non-structural 5 gene. The GenBank accession number, virus name, origin, collection country and year are noted. The JEV and ISFV sequences obtained in this study are marked in red. The numbers above each branch represent the bootstrap support for the Bayesian analyses, maximum likelihood, and neighbor-joining, respectively, based on 1000 replicates. The scale-bar indicates 0.1 substitutions per site. *AAFV* Anopheles-associated flavivirus, *AeFV* Aedes flavivirus, *AnFV* Anopheles flavivirus, *CFAV* cell fusing agent virus, *CHAOV* Chaoyang virus, *CTFV* Culex theileri flavivirus, *CxFV* Culex flavivirus, *DGV* Donggang virus, *HANKV* Hanko virus, *JEV* Japanese encephalitis virus, *KRV* Kamiti River virus, *LAMV* Lammi virus, *LTNV* La Tina virus, *MMLV* Montana myotis leukoencephalitis virus, *MMV* Marisma mosquito virus, *MODV* Modoc virus, *NAKV* Nakiwogo virus, *PAFV* Phlebotomus associated flavivirus, *QBV* Quang Binh flavivirus, *OcFV* Ochlerotatus caspius flavivirus, *SLEV* Santa Louis encephalitis virus, *TBEV* tick-borne encephalitis virus, *WNV* West Nile virus, *YDFV* Yamadai flavivirus, *YNCxFV* Yunnan Culex flavivirus

**Fig. 3:**
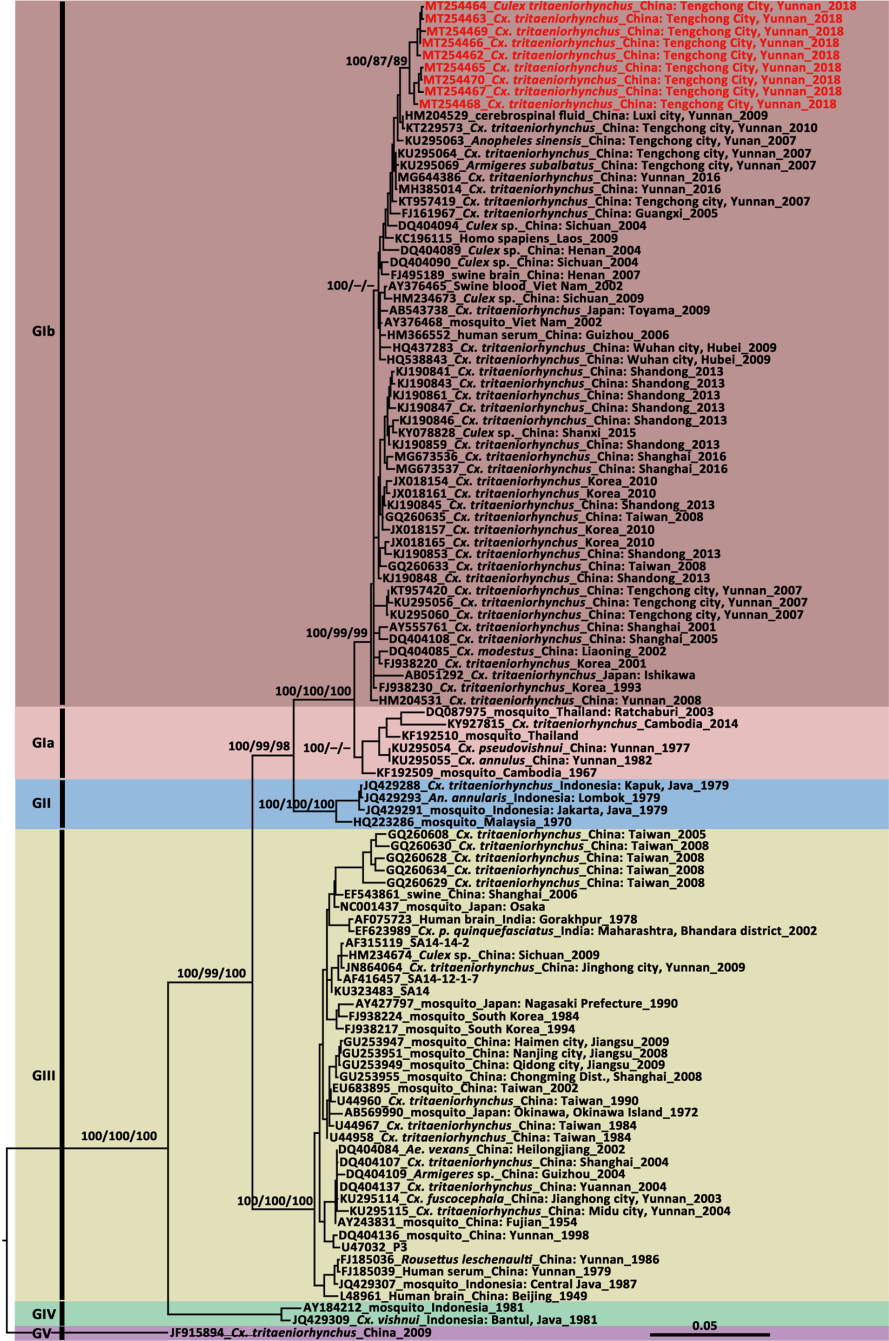
Phylogenetic tree generated by Bayesian analysis of Japanese encephalitis virus envelop gene sequences. The GenBank accession number, origin, collection country and year of each strain are noted. The JEV sequences obtained in this study are marked in red. The numbers above each branch represent the bootstrap support of the Bayesian analyses, maximum likelihood, and neighbor-joining, respectively, based on 1000 replicates. The scale-bar indicates 0.05 substitutions per site. Sequences shaded tan represent the GI-a genotype, those shaded rose-brown represent the GI-b genotype, those shaded sky blue represent the GII genotype, those shaded khaki represent the GIII genotype, those shaded aquamarine represent the GIV genotype, and those shaded purple represent the GV genotype

The integrity of RNA extracted from the mosquito pools was tested by the amplification of the *18S* rRNA. This amplification was successful in all the 354 pools. In further pathogen detection, 22 flavivirus strains were recovered by the successful amplification of the partial *NS5* gene. According to the phylogenetic analysis (Fig. 2), JEVs were present in 15 pools of *Cx. tritaeniorhynchus*. The *prM* and *E* genes were successfully amplified in 12 and 9 of the 15 JEV-positive pools, respectively. Phylogenetic analyses (Fig. 3) indicated that GI-b was the only genotype detected among samples collected in Tengchong in 2018. The species name, collection information, host species and GenBank accession numbers of JEV and insect-specific flavivirus (ISFV) strains obtained in this study, are shown in Table 1. No sequences ascribable to the PCR target were obtained using the PCR for alphaviral and orthobunyaviral genomes. Moreover, no *Plasmodium* spp. was found in this study. Particularly, in the partial *NS5* gene amplification, a flavivirus-positive pool of *An. sinensis* showed 93.5% similarity to the 12R (MN090154) strain of Flaviviridae sp. from *Phlebotomus* sp. collected in Bosnia and Herzegovina in 2018. This putative new ISFV had not been named yet. We designated the new virus as Anopheles-associated flavivirus (AAFV) tentatively.

**Table 1:** Details of flavivirus strains detected from mosquitoes, captured in Tengchong from July to October in 2018

Virus	Strain	Host	Collection date	Geographical location	Habitat	GenBank ID		
						***NS5***	***E***	***prM***
JEV	TC2G5_18-8E-Y–T-Cxt-Y-5–19	*Culex tritaeniorhynchus*	6-Aug-2018	Fuyu village, Beihai township	Cattle farm	MT254428	MT254462	MT254450
JEV	TC2F12_18-8E-Y–T-Cxt-Y-5–30	*Cx. tritaeniorhynchus*	6-Aug-2018	Fuyu village, Beihai township	Cattle farm	MT254429		
JEV	TC2F6_18-8E-Y–T-Cxt-Y-5–35	*Cx. tritaeniorhynchus*	6-Aug-2018	Fuyu village, Beihai township	Cattle farm	MT254430	MT254463	MT254451
JEV	TC2H11_18-8E-Y–T-Cxt-Y-5–44	*Cx. tritaeniorhynchus*	6-Aug-2018	Fuyu village, Beihai township	Cattle farm	MT254431	MT254464	MT254452
JEV	TC2A1_18-8 M-Y–T-Cxt-Y-5–8	*Cx. tritaeniorhynchus*	14-Aug-2018	Fuyu village, Beihai township	Cattle farm	MT254432		
JEV	TC2B4_18-8 M-Y–T-Cxt-Y-5–10	*Cx. tritaeniorhynchus*	14-Aug-2018	Fuyu village, Beihai township	Cattle farm	MT254433	MT254465	MT254453
JEV	TC1H10_18-8 M-Y–T-Cxt-Y-5–11	*Cx. tritaeniorhynchus*	14-Aug-2018	Fuyu village, Beihai township	Cattle farm	MT254434	MT254466	MT254454
JEV	TC2B10_18-8 M-Y–T-Cxt-Y-5–21	*Cx. tritaeniorhynchus*	14-Aug-2018	Fuyu village, Beihai township	Cattle farm	MT254435		MT254455
JEV	TC1H8_18-8 M-Y–T-Cxt-Y-5–25	*Cx. tritaeniorhynchus*	14-Aug-2018	Fuyu village, Beihai township	Cattle farm	MT254436		MT254456
JEV	TC4B3_18-9E-Y–T-Cxt-Y-5–7	*Cx. tritaeniorhynchus*	5-Sep-2018	Fuyu village, Beihai township	Cattle farm	MT254437		
JEV	TC4E10_18-9E-Y–T-Cxt-Y-5–11	*Cx. tritaeniorhynchus*	5-Sep-2018	Fuyu village, Beihai township	Cattle farm	MT254438	MT254467	MT254457
JEV	TC4C11_18-9E-Y–T-Cxt-Y-5–12	*Cx. tritaeniorhynchus*	5-Sep-2018	Fuyu village, Beihai township	Cattle farm	MT254439	MT254468	MT254458
JEV	TC4E7_18-9E-Y–T-Cxt-Y-5–21	*Cx. tritaeniorhynchus*	5-Sep-2018	Fuyu village, Beihai township	Cattle farm	MT254440		MT254459
JEV	TC4D5_18-9E-Y–T-Cxt-Y-5–28	*Cx. tritaeniorhynchus*	5-Sep-2018	Fuyu village, Beihai township	Cattle farm	MT254441	MT254469	MT254460
JEV	TC4A5_18-9L-Y–T-Cxt-Y-5–1	*Cx. tritaeniorhynchus*	27-Sep-2018	Fuyu village, Beihai township	Cattle farm	MT254442	MT254470	MT254461
YNCxFV	TC2H8_18-8E-Y–T-Cxt-Y-5–17	*Cx. tritaeniorhynchus*	6-Aug-2018	Fuyu village, Beihai township	Cattle farm	MT254443		
YNCxFV	TC4C9_18-9E-Y–T-Cxt-Y-5–18	*Cx. tritaeniorhynchus*	5-Sep-2018	Fuyu village, Beihai township	Cattle farm	MT254444		
CTFV	TC2B9_18-8 M-Y–T-Cxv-Y-5–1	*Cx. vagans*	14-Aug-2018	Fuyu village, Beihai township	Cattle farm	MT254445		
AeFV	TC4E2_18-9E-Y–T-Aea-B-JM-1	*Aedes aegypti*	5-Sep-2018	Changdong community, Tengyue town	Suburb residential area	MT254446		
AeFV	TC4A8_18-9L-Y–T-Aea-B-1–1	*Ae. aegypti*	27-Sep-2018	Changdong community, Tengyue town	Suburb residential area	MT254447		
YDFV	TC2A6_18-8 M-Y–T-Anp-Y-2–1	*Cx. pseudovihnui*	14-Aug-2018	Tiancheng community, Tengyue town	Hospital	MT254448		
ISFV	TC3D4_18-7 M-Y–T-Ans-Y-5–11	*Anopheles sinensis*	17-Jul-2018	Fuyu village, Beihai township	Cattle farm	MT254449		

*AeFV* Aedes flavivirus, *CTFV* Culex theileri flavivirus, *JEV* Japanese encephalitis virus, *NS5* non-structural 5 gene, *E* envelope gene, *prM* pre-membrane gene, *YDFV* Yamadai flavivirus, *YNCxFV* Yunnan Culex flavivirus

#### The whole genome of JEV and AeFV

The length of the whole-genome of the newly detected JEV (TC4E10_18-9E-Y–T-Cxt-Y-5–11 strain MT254426) was 10 953 bp, with an open reading frame coding for 3432 amino acids flanked by 96 and 573 nucleotides at the 5′UTR and 3′UTR ends, respectively. The complete genome was amplified using a total of 11 pairs of overlapping primers (Additional file 1: Table S1). The length of the whole-genome of newly detected AeFV (TC4E2_18-9E-Y–T-Aea-B-JM-1 strain, MT254427) was 10 998 bp, with an open reading frame coding for 3341 amino acids flanked by 96 and 909 nucleotides at the 5′UTR and 3′UTR ends, respectively. The complete genome was amplified using a total of ten pairs of overlapping primers (Additional file 1: Table S2).

### Molecular characterization and phylogenetic analysis of JEV

#### Phylogenetic analyses on NS5, prM, and E gene of JEV.

The phylogenetic tree based on the *NS5* gene (Fig. 2) showed that the genus of Flaviviridae contains four main clusters, including mosquito-borne flaviviruses (the majority are pathogenic), tick-borne flaviviruses, no-known vector flaviviruses and classical ISFVs. The ISFVs are divided into classical ISFVs and dual-host affiliated ISFVs. The dual-host affiliated ISFVs are phylogenetically close to the mosquito-borne pathogenic flaviviruses than the clade of classical flaviviruses. Fifteen flavivirus-positive sequences, belonging to the mosquito-borne flavivirus, were clustered in the JEV clade, and the other seven sequences were scattered in classical ISFV clusters.

The topologies of the trees produced from the JEV *E* (Fig. 3) and *prM* genes (Additional file 1Fig. S1) identified five major clades corresponding to genotypes I, II, III, IV and V. Moreover, GI was composed of two distinct clades, representing the two sub-genotypes, GI-a and GI-b. Based on the supporting values of the three phylogenetic trees, for the majority of lineages, the Bayesian method resulted in relatively higher bootstrap values than the ML and NJ methods. Henceforth, the Bayesian tree is presented here with the supporting values of all the three methods. The *prM* (580 nt) tree showed lower bootstrap values but displayed a topology consistent with that obtained with the *E* gene sequence (1500 nt).

The *prM* gene sequences of the JEV strains from Tengchong County showed high levels of similarity with each other at the nucleotide (range: 97.8–100%) and amino acid (range: 95.9–100%) levels, but a lower homology to the vaccine SA14-14-2 strain (87.8–88.7% at the nucleotide level and 94.3–96.9% at the amino acid level). For the *E* gene, 98.9–100% nucleotide sequence identity and 99.6–100% amino acid sequence identity was observed among the newly detected Tengchong strains, while they were 87.2%–87.8% and 96.7%–97.0% similar regarding the nucleotide and amino acid levels when compared with the vaccine strain SA14-14-2. Amino acid substitutions unique to the newly sequenced strains and those different from their closest sequences are shown in Additional file 1: Table S3. In the *E* gene tree (Fig. 3), the newly obtained strains fell into the GI-b cluster and were most closely related to the strain obtained in Tengchong in 2010 (DH10M978 strain, KT229573), but far from several strains circulated in Tengchong in 2007.

## Comparison on deduced amino acid of E protein among JEV strains

Deduced amino acid differences in E protein sequences were aligned for comparison among the newly sequenced strains and the vaccine strain (SA14-14–2) currently used in China. Six amino acid residues in the newly detected Shanghai JEV strains differed from those in the SA14-14–2-derived strain (SA14): E123 (Ser → Asn), E129 (Thr → Met), E222 (Ala → Ser), E327 (Ser → Thr), E366 (Ala → Ser), E433 (Val → Ile), no differences were observed in key amino acid sites related to antigenicity.

We compared the deduced amino acid sequences of the whole-genome between TC4E10_18-9E-Y–T-Cxt-Y-5–11 and DH10M978 strain, and there were four non-synonymous changes distributed in each capsid (C, ^127^Thr → Ala), pre-membrane (prM, ^128^Met → Ile), E (^727^Val → Leu), and non-structure 1 (NS1, ^822^Trp → Cys) proteins. Seventy-eight substitutions were observed between the TC4E10_18-9E-Y–T-Cxt-Y-5-11 strain and the SA14-14-2 strain (Additional file 1: Table S3), distributed in C (5 positions), prM (5 positions), E (15 positions), NS1 (15 positions), NS2A (6 positions), NS2B (4 positions), NS3 (8 positions), NS4A (6 positions), and NS5 (14 positions) proteins. No change was detected in the NS4B region of the viral protein. Among TC4E10_18-9E-Y–T-Cxt-Y-5-11, DH10M978, SA14-14-2, and SA strains, three amino acid substitutions were found to be unique in the TC4E10_18-9E-Y–T-Cxt-Y-5–11 strain, including ^128^Met → Ile, ^727^Val → Leu, and ^822^Trp → Cys.

### Sequence analysis and phylogenetic characterization of ISFVs

Sixty-eight partial *NS5* sequences of 30 representative flavivirus that were retrieved from GenBank and the 22 described here were aligned. As shown in Fig. 2, all the lineages, including individuals representing the same ISFV, formed a distinct lineage with a high bootstrap value. The lineages were host-specific in general and the seven newly obtained ISFVs from Tengchong were distributed in five lineages.

The Yamadai flavivirus (YDFV) was detected in *Cx. pseudovihnui* (MT254448), distributed in Tiancheng Community, Tengchong County. Analysis of their partial *NS5* sequence fragments revealed 91.5–91.9% similarity with the YDFVs sequences (LC513840, and AB981186) from *Cx. tritaeniorhynchus* collected from Japan that is available in GenBank. Two strains of Yunnan Culex flavivirus (YNCxFV, MT254443, and MT254444) were found in two pools of *Cx. tritaeniorhychus*, sharing 92.4% sequence similarity. Based on the short *NS5* sequence fragments, the nucleotide similarity between the newly obtained YNCxFV strains and the YNCxFV from Lushui County, Yunnan Province (NC_021069) was 91.5–95.0%, while it was 81.9–87.7% with QBVs available in GenBank. In the *NS5* tree (Fig. 2), the YNCxFV sequences formed a sister lineage to the group containing QBV sequences with a moderate bootstrap value (64% by Bayesian method). A Culex theileri flavivirus (CTFV) strain was detected in a pool of *Cx. vagans*. It shares 90.8% similarity in nucleotide sequence with the CTFV strain from Portugal (HE574573). In the *NS5* tree, several sequences, previously identified as Culex flavivirus (CxFV) (MH827522, MF582373, MF582377, and MF582380) and mosquito flavivirus (KY247170, KX652377, and KX652378) were gathered in the CTFV lineage. The new CTFV stain shared 90.2–92.3% nucleotide sequence similarity with other CTFV sequences. The CTFV lineage was close with the cluster, which was combined with lineages of YNCxFVs, YDFVs, and QBVs. Two *Ae. albopictus* pools were positive for AeFV. They (MT254446, and MT254447) shared 98.9% nucleotide sequence similarity and were most related to La Tina virus (KY320649) with 96.9–97.3% similarity. They were gathered with other AeFVs in the *NS5* tree. A new detected flavivirus strain from a pool of *An. sinensis* shared 93.5% similarity with the Flaviviridae sp. from *Phlebotomus* sp. (MN090154) in 157 nt of *NS5* gene, and lower than 69.6% nucleotide sequence similarity and 71.3% amino acid sequence similarity with its most similar mosquito flavivirus isolate OccaFV3 (GQ476995). In the phylogenetic tree, the two strains (MT254449, and MN090154) formed a monophyletic lineage, supported by high bootstrap value, and distant from other Anopheles specific flaviviruses. Their nucleotide sequence similarity with other ISFVs was below the threshold proposed by Kuno et al. [56], who defined the species as a class of viruses with a nucleotide sequence similarity higher than 84%. These results suggest that AAFV is probably a new member of the ISFV family.

### The infection rate of flavivirus in culicines

All the JEV-positive sequences were obtained from *Cx. tritaeniorhynchus* pools. The overall infection rate (Table 2) according to bias-corrected MLE and MIR of JEV in *Cx. tritaeniorhynchus*, with 95% confidence intervals (*CI*) were 2.4 (1.4–3.9) and 2.3 (1.1–3.5) per 1000 individuals, respectively. JEVs were detected in samples collected in August and September, and no JEV-positive pools were found in July and October. The bias-corrected MLE of JEV infection rates were 3.0 (1.5–5.6), and 3.9 (1.6–8.2) per 1000 *Cx. tritaeniorhynchus* in August and September, respectively. The bias-corrected MLE of YNCxFV, YDFV, AeFV, and AAFV, expressed as the number of infected mosquitoes per 1000 individuals, for *Cx. tritaeniorhynchus*, *Cx. pseudovihnui*, *Ae. albopictus*, and *An. sinensis* were 0.3 (0.1–1.0), 7.6 (1.4–69.3), 41.4 (9.3–127.0), and 0.4 (0.0–1.7), respectively.

**Table 2:** Bias Corrected Maximal likelihood estimation (MLE) and minimum infection rate (MIR) of flaviviruses during mosquito activity season of Tengchong in 2018

Detected virus	Host	No. of individuals	No. of positive pools	No. of pools	Positive pool rate (%)	Bias corrected MLE (95% *CI*)	MIR (95% *CI*)
JEV	*Culex tritaeniorhynchus*	6490	15	149	10.07	2.44 (1.43–3.94)	2.31 (1.14–3.48)
YNCxFV	*Cx. tritaeniorhynchus*	6490	2	149	1.34	0.31 (0.06–1.01)	0.31 (0.00–0.74)
AeFV	*Aedes albopictus*	43	2	7	28.57	41.43 (9.30–127.70)	46.51 (0.00–109.46)
YDFV	*Cx. pseudovihnui*	50	1	2	50.00	7.62 (1.35–69.27)	20.00 (0.00–58.81)
AAFV	*Anopheles sinensis*	2775	1	145	0.69	0.36 (0.02–1.74)	0.36 (0.00–1.07)

*AeFV* Aedes flavivirus, *CI* confidence interval, *JEV* Japanese encephalitis virus, *YDFV* Yamadai flavivirus, *YNCxFV* Yunnan Culex flavivirus

## Discussion

The newly detected JEV Tengchong strains showed low genetic diversity in *NS5*, *prM*, and *E* genes, and were most closely related to the strain from the local area in 2010, suggesting that frequent transmission of JEV GI-b sub-genotype occurred in Tengchong. The GI genotype was isolated from Yunnan in 1977 and 1982 [57]. Considering the *E* gene tree, these belonged to the GI-a sub-genotype (Fig. 3). However, the GI genotype had not been detected in Yunnan since then until GI-b sub-genotype was isolated in 2007 [58]. The GI-a sub-genotype was once prevalent in tropical Asia in the 1970s and 1980s [57, 59]. Few strains of the GI-a sub-genotype has been detected after that. Recently, it has been detected in humans (2015) and mosquitoes (2014) in Cambodia [60]. In addition, the GIII circulated in Yunnan between 1978 and 2004 as the predominant genotype but has not been found in recent years [58]. This coincides with the replacement of GIII of JEV by GI in several Asian countries during the past few decades [61, 62], whereas GIII was found using a neutralization test among the laboratory-confirmed JE cases in Myanmar in 2013 [63]. Further clarification using additional samples is required to determine if GIII co-circulates with GI in Tengchong.

There were no corresponding mutations at the E107, E138, E176, E177, E264, E279, E315 or E439 loci of the newly obtained strains, which are related to virus attenuation [54]. However, six amino acid residues in the newly detected JEV Tengchong strains differed from those in the vaccine SA14-14-2-derived strain. Also, the co-evolution of two pairs of sites within GIs (residues S89N to F360Y and M129T to I141V) was not observed in the new Tengchong strains, in which these residues functionally interact with each other to maintain a functional E protein [61]. Twelve combinations of amino acids, with SKSS and NKSS as dominant ones, were observed at four sites in the E protein (E123, E209, E227 and E408), which were defined based on the predicted positive selections [64]. NKSS is the sole haplotype presented in the newly obtained strains. SKSS haplotype was observed in strains collected in 2007 from Tengchong (KT957420, KU295056, and KU295060). Notably, more JEV sequences involved in recent JEV outbreaks [7, 65, 66] contain the SKSS haplotype.

The annual morbidity and mortality of JE were 0.75/100 000, and 5.6%, respectively, in Yunnan Province, with an increase in adult JE incidences [67]. The introduction of JE vaccines in China since the 1970s, and the following intervention of intensive JE vaccine program in children have dramatically decreased JE cases [68]. However, JE outbreaks occurred occasionally in China [5–8] and other countries in Southeast Asia [69, 70], with the increase in adult cases. Irrigated rice production and pig rearing are the two main factors inducing the circulation of JEV in nature. In this study, all the JEV-positive pools were collected in Fuyu Village, mainly in August and September in 2018, coinciding with the peak of the JE incidence in Yunnan [67]. The bias-corrected MLE of JEV infection rates in September reached 3.9 with 8.2 as the upper limit per 1000 individuals. The infection rate was high in the courtyards of farmers’ households with pigsties (7.4 per 10 000) in Shanxi, which is located in the epidemic area of adult JE cases [5, 66]. The MLE of JEV was 11.8 per 1000 *Cx. tritaeniorhynchus* during the 2010 JE outbreak in Korea [71]. The prevalence of flavivirus, e.g. WNV (data unavailable for JEV) that represents an “epidemic risk” is more than 5 per 1000 mosquitoes [7], indicating that the Fuyu Village has a potential epidemic risk. The aerial view of the Fuyu Village showed the village to be characterized by a rural environment with dense housing, with a large paddy field nearby (Fig. 1b). The collection site of flavivirus-positive pools was a cattle farm, but domestic pigpens were distributed sporadically nearby. In addition, there was a pig farm 2 km away from the cattle farm. The natural and social environment was suitable for the transmission of JEV in the Fuyu Village. Notably, cattle are dead-end host, and *Culex* mosquitoes preferentially feed on cattle. The cattle can be used to divert infected mosquitoes from humans and swine [72]; however, it reflects JEV prevalence in the local area.

As JE is under the umbrella of the vaccine program, the JEV vaccine, included in the Expand Program of Immunization in China since 2008, free of charge, was implemented in children with a coverage of almost 100% in Tengchong. However, there exists the possibility that for a few infants, the immunization time was postponed, as their parents worked abroad. From 2017 to 2019, JE clinical cases were not observed in Tengchong reported by the officials at the Tengchong County Disease Prevention and Control. However, we cannot exclude the possibility of JE cases with asymptomatic or mild symptoms, since JEV infection is symptomatic in less than 1% of the cases [73]. Considering the high infection rate of JEV in mosquitoes, it is wise to carry out a baseline survey to detect the coverage of JEV antibodies in the serum of local residences, who are lacking vaccination. The vaccination strategy in adults could be established and conducted according to the infectious risk estimated by the results of the baseline survey. Moreover, segregation of livestock farms away from human residential areas is suggested to prevent the spread of JEV from animal reservoir to human population. Besides, the surveillance on the dynamics of JEV infection in mosquitoes is essential to predict the epidemic risk of JEV outbreak in the local area. Moreover, with the promotion of the Belt and Road Initiative, imported cases—following the increase in border business trade and travel—should be monitored more closely to prevent secondary incidences (the infection of a local population from an imported case).

ISFVs are a type of flavivirus, propagating only in mosquito cells, unable to replicate in mammalian cells [74]. Several novel flaviviruses have been isolated and characterized as ISFVs in this century, and documented around the globe [38, 75–80]. To our knowledge, there is no record of the presence and diversity of ISFVs in Tengchong. Infection with CxFV can increase the insect’s vector competence for WNV, as reported in field and laboratory tested *Cx. tritaeniorhynchus* [81, 82]. Other studies showed that earlier infection of ISFVs suppresses subsequent replication of mosquito-borne flaviviruses associated with human diseases [83, 84]. Five kinds of ISFVs were found in Tengchong, and no pools were co-infected with both ISFVs and JEVs, implying possibility of the existence of superinfection exclusion between ISFVs and vertebrate-infecting flaviviruses. However, all JEV-positive pools of *Cx. tritaeniorhynchus* contained YDFV in Yamaguchi, Japan, in 2013 [85]. Thus, more evidence is required to address whether ISFV-infected mosquitoes can escape vector competence or superinfection exclusion for pathogenic flavivirus infections.

In general, ISFVs are the host genus or species-specific by vertical transmission [86]. YDFV were previously isolated from Japan in pools of *Cx. tritaeniorhynchus* with the infection rate of 1.8 per 1000 individuals (MIR) [85]. Whereas, it was present in one of two pools of *Cx. pseudovihnui*, and absent in *Cx. tritaeniorhynchus* pools. In addition, the CTFV was firstly isolated from samples collected from *An. vagus* pool in Indonesia, collected in 1981 [87]. Recently, it was also found in Guangdong Province, China ([88], *Cx. quinquefasciatus*, 2013), Myanmar ([89], *Cx. tritaeniorhynchus*, *Cx. vishnui*, *Cx. fuscocephalus*, 2014), and Turkey ([90], *Cx. theileri*, 2015); it was detected in a pool of *Cx. vagans* in Tengchong, China. Interestingly, CTFV was previously identified in *Cx. tritaeniorhynchus* samples, collected in Portugal, in 2009 [91]. This indicates that the ISFVs have probably circulated in nature for a long time, and have wide geographic and host ranges, although with a short history of discovery. The new YNCxFV strain from one pool of *Cx. tritaeniorhynchus* was closely related to the other two YNCxFV strains (MT254443, and MT254444), from Lushui County, approximately 180 km away from Tengchong. To date, these were the only three strains of YNCxFV available on GenBank, and no other records were reported in other places, except the Yunnan Province. AeFVs were detected in two out of seven *Ae. albopictus* pools with the infection rate of 41.4 per 1000 individuals (bias-corrected MLE). It was higher than what was detected in the Songjiang District, Shanghai, in 2016 (33.9 per 1000 individuals) [38]. The AAFV from *An. sinensis* were far distant from the lineage of AnFVs. The other strain (MN090154) was isolated from *Phlebotomus* sp. in Bosnia and Herzegovina, collected in the same year. Precisely 92.9% nucleotide similarity and 100% amino acid similarity were observed between the two strains in the highly conserved *NS5* gene, and below the cutoff value (84%) among species of flavivirus with their close relatives (OcFVs), which was 69.6%. It’s a pity that we failed to amplify longer sequences in *NS5* and *E* genes of AAFV. Moreover, the most diverse and prevalent ISFV, CxFV, mainly hosted in *Cx. pipiens* complex, was not detected in *Cx. pipiens quinquefasciatus* in this study. The CxFVs can be divided into two genotypes. One is prevalent in Asia and the United States and is mainly detected in *Cx. pipiens pipens*, the other is distributed in Africa, the Caribbean, and Latin America, sharing the same host *Cx. pipiens quinquefasciatus* [92]. The latter was found in the Middle East region recently, while the genotype of CxFV in *Cx. pipiens quinquefasciatus* populations in Asia has not been found yet.

The main limitation of our study is that the supernatants of mosquito homogenate viruses were not cultivated in mosquito or human cell lines. The low concentration or titer of a given virus in mosquito homogenates may have influenced the results of pathogen detection and amplification of long sequence. Additional virus-positive pools might have been missed by direct RNA isolation and thus, the actual infection rate of arboviruses in Tengchong may be underestimated. Furthermore, as the JE potential epidemic focus we detected here was in a cattle farm, which is the dead-end host, we need to analyze arbovirus in mosquitoes collected in a pig farm near the cattle farm to see the infection rate of JEV in the amplifying host. Also, the taxonomy of the tentatively named AAFV has not been resolved. The evidence proposing AAFV as a novel species of ISFV was not sufficient. It is crucial to isolate the virus in cell culture, obtain its whole-genome, electron microscopy of virion particles, the cytopathic effect, and perform neutralization tests to clarify the taxonomic status of the species further.

## Conclusions

A potential JE epidemic focus was detected with the high JEV infection rate in the field-caught mosquitoes in Tengchong. The phylogenetic analyses showed that the 15 strains of newly detected JEV belong to the GI-b genotype, similar to the strain found in Tengchong in 2010. It indicates a possible JEV epidemic in the local area. Notably, all the JEV-positive pools were collected in a cattle farm in the Fuyu Village, which is surrounded by dense residential housing, domestic pigpens, and intensified irrigated rice paddies. It is necessary to implement the immunization program for children persistently, and investigate the number of adults who are not inoculated with the JE vaccine in local area. In addition, moving all domestic pigs into a communal piggery at a location more than 5 km away from the human population could help reduce the risk of JE transmission to humans. Moreover, five species of ISFVs, AeFV, CTFV, YDFV, YNCxFV, and AAFV were found co-circulating in Tengchong. This is the first record of ISFVs in this area. The results strongly suggest the need for widespread and sustained mosquito-based arbovirus surveillance in local areas. It allows accurate and timely estimations of the actual disease burden and prevalence of JE and other mosquito-borne pathogens.

## Supplementary Information

**Additional file 1: Fig. S1.** Phylogenetic tree generated by Bayesian analysis of Japanese encephalitis virus pre-membrane gene sequences; **Table S1.** Primer sets used for sequencing the JEV TC4E10_18-9E-Y–T-Cxt-Y-5–11 strain; **Table S2**. Primer sets used for sequencing the AeFV TC4A8_18-9L-Y–T-Aea-B-1–1 strain; **Table S3.** Comparison on amino acid substitutions among JEV TC4E10_18-9E-Y–T-Cxt-Y-5–11 strain, DH10M978 strain, vaccine strain SA14-14–2, and SA14 strain.

## Data Availability

All data generated or analysed during this study are included in the published article and Additional file.

## Abbreviations

AAFV: Anopheles-associated flavivirus
AeFV: Aedes flavivirus
AnFV: Anopheles flavivirus
*CI*: Confidence interval
CTFV: Culex theileri flavivirus
CxFV: Culex flavivirus
*E*: Envelope gene
JEV: Japanese encephalitis virus
*NS5*: Non-structural 5 gene
PAFV: Phlebotomus associated flavivirus
*prM*: Pre-membrane gene
QBV: Quang Binh flavivirus
OcFV: Ochlerotatus caspius flavivirus
WNV: West Nile virus
YDFV: Yamadai flavivirus
YNCxFV: Yunnan Culex flavivirus
