# Supplementary material for: Molecular epidemiology of mosquito-borne viruses at the China–Myanmar border: discovery of a potential epidemic focus of Japanese encephalitis
Source: Infect Dis Poverty. 2021 Apr 26;10:57. doi: 10.1186/s40249-021-00838-z (PMC8073957; doi:10.1186/s40249-021-00838-z)
Supplement: Supplementary file 1 — Additional file 1: Fig. S1. Phylogenetic tree generated by Bayesian analysis of Japanese encephalitis virus pre-membrane gene sequences; Table S1. Primer sets used for sequencing the JEV TC4E10_18-9E-Y–T-Cxt-Y-5–11 strain; Table S2. Primer sets used for sequencing the AeFV TC4A8_18-9L-Y–T-Aea-B-1–1 strain; Table S3. Comparison on amino acid substitutions among JEV TC4E10_18-9E-Y–T-Cxt-Y-5–11 strain, DH10M978 strain, vaccine strain SA14-14–2, and SA14 strain. [file 40249_2021_838_MOESM1_ESM.docx]

**
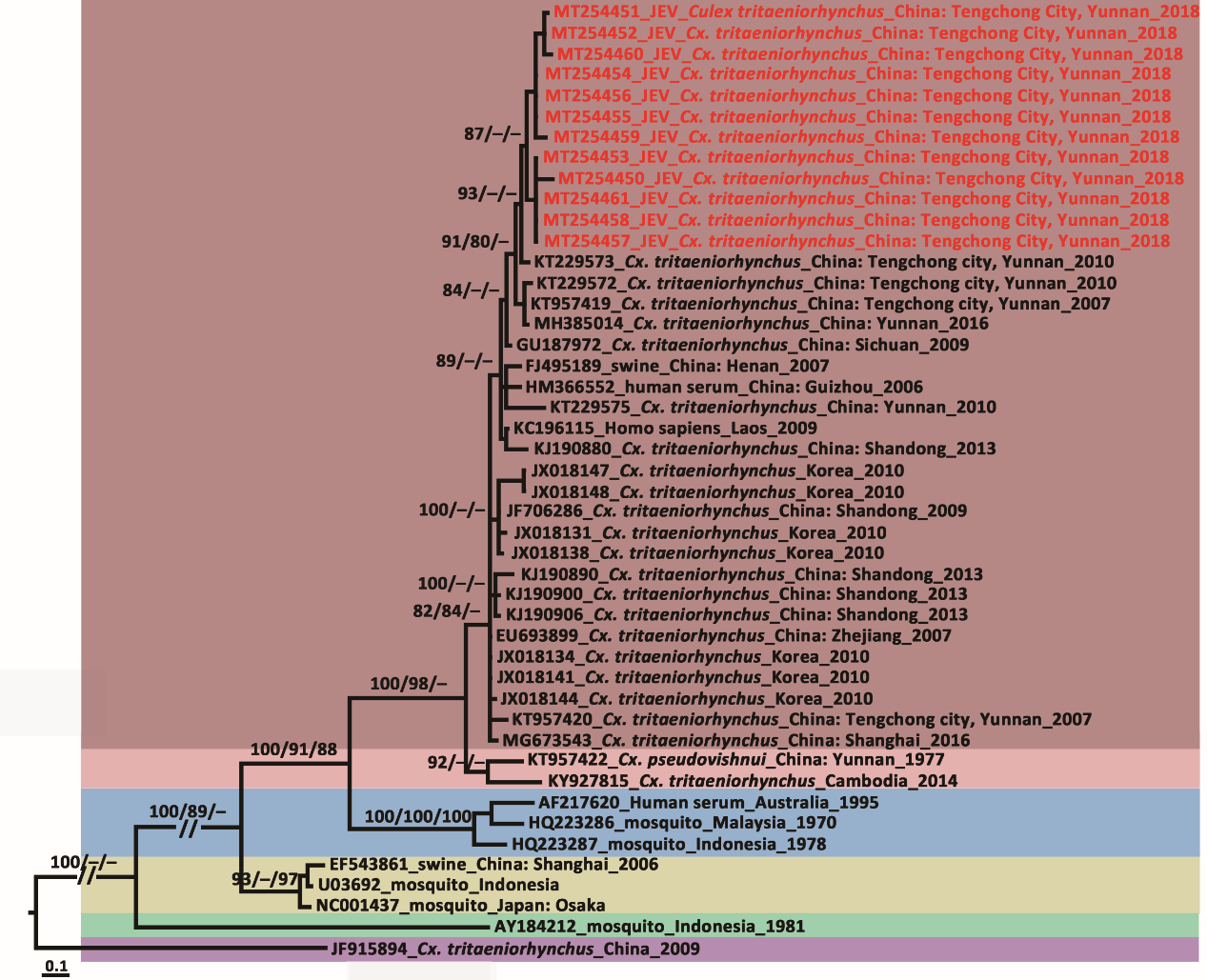
**

**Fig. S1 Phylogenetic tree generated by Bayesian analysis of Japanese encephalitis virus pre-membrane gene sequences.** The GenBank accession number, origin, collection country and year of each strain are noted. The JEV sequences obtained in this study are marked in red. The numbers above each branch represent the bootstrap support of the Bayesian analyses, maximum likelihood, and neighbor-joining, respectively, based on 1,000 replicates. The scale-bar indicates 0.1 substitutions per site. Sequences shaded tan represent the GI-a genotype, those shaded rose-brown represent the GI-b genotype, those shaded sky blue represent the GII genotype, those shaded khaki represent the GIII genotype, those shaded aquamarine represent the GIV genotype, and those shaded thistle represent the GV genotype.

**Tab. S1 Primer sets used for sequencing the JEV TC4E10_18-9E-Y-T-Cxt-Y-5-11 strain**

| Primer | Position | Sequence, 5’-3^’a^ | Polarity |
| --- | --- | --- | --- |
| 1-F | 1–27 | AGAAGTTTATCTGTGTGAACTTCTTGG | Sense |
| 1-R | 1156–1177 | CAGTGACTGAAGCGTGATAGCA | Reverse |
| 2-F | 962–982 | TCGCTCCGGCTTACAGTTTTA | Sense |
| 2-R | 2012–2032 | CCACAGGGGTCATGTCGTTTA | Reverse |
| 3-F | 1849–1868 | ATGGACAAACTGGCCCTGAA | Sense |
| 3-R | 2950–2967 | TGTGATGCCGAAGCCGAA | Reverse |
| 4-F | 2755–2774 | CCTGTGGGGAGATATCGGTC | Sense |
| 4-R | 3885–3908 | ACTCCGATCTGTAAATCCACTGAA | Reverse |
| 5-F | 3754–3775 | GTTCTGCACCTCGCTCTGATAG | Sense |
| 5-R | 4919–4942 | TTGTCTGGATGTTTACTGCAGGTT | Reverse |
| 6-F | 4740–4760 | TGTTTTCCACACATTGTGGCA | Sense |
| 6-R | 5983–6004 | GATTCCTGCCCACTCTACCTCT | Reverse |
| 7-F | 5808–5831 | TGGAGATTGGGACTTTGTTATCAC | Sense |
| 7-R | 7091–7113 | TGTGGTGACGTATTCTGAGGTGA | Reverse |
| 8-F | 6979–6997 | GCACCAGGACTGACCGGAT | Sense |
| 8-R | 8210–8232 | GCAGAGCACTTTTATGCAGAACT | Reverse |
| 9-F | 8027–8048 | AGAGTTACGGTTGGAACCTGGT | Sense |
| 9-R | 9301–9324 | CAAGTCGGTTCTAGTGATTCTGGT | Reverse |
| 10-F | 9069–9089 | CGGAGAGTTCGGAAAGGCTAA | Sense |
| 10-R | 9664–9683 | TCGTCTCCACTGATCGCCAT | Reverse |
| 11-F | 9508–9530 | GCTCTCAACACATTCACGAACAT | Sense |
| 11-R | 10945–10965 | AGATCCTGGTGGTTTCCTCAC | Reverse |

^a^The primers were designed from the strain DH10M978 (KT229573).

**Tab. S3 Primer sets used for sequencing the AeFV TC4A8_18-9L-Y-T-Aea-B-1-1 strain**

| Primer | Position | Sequence, 5’-3^’a^ | Polarity |
| --- | --- | --- | --- |
| 1-F | 1–24 | TCCCTAGTTGGATTACACCAATTT | Sense |
| 1-R | 1244–1263 | AACAAGCACGGATACATTTACCA | Reverse |
| 2-F | 1082–1101 | AAGACACATGCCCTGGTGGA | Sense |
| 2-R | 2285–2305 | AGGCATAATCGTCGAGCTCAA | Reverse |
| 3-F | 2104–2126 | ATTGTTTTCCTTGGTAGAGGGGT | Sense |
| 3-R | 3297–3317 | TCCATTAGCGGCTCTTCTTCA | Reverse |
| 4-F | 3101–3123 | ATCCAAAGTGTGACGGTAGAATG | Sense |
| 4-R | 4412–4431 | CAGGTCATCCGAGCGACAAT | Reverse |
| 5-F | 4227–4243 | TAGCGATGAGGGAATCACTATTG | Sense |
| 5-R | 5394–5412 | GAATGCGATGGCTTGGTCA | Reverse |
| 6-F | 5259–5280 | TCACTTCATGGATCCCATGTCA | Sense |
| 6-R | 6387–6408 | GGAATATGACACGAGGCAGGTA | Reverse |
| 7-F | 6223–6245 | CATGGAATTTGGAAAAAGACGAC | Sense |
| 7-R | 7338–7362 | AAACAGGAAGTTCATGTCAATCAAT | Reverse |
| 8-F | 7143–7165 | GTCTGTTGACAACACTCGAGCTG | Sense |
| 8-R | 8329–8351 | TGTCCTCCGACTTTTACGTCATC | Reverse |
| 9-F | 8174–8192 | GAACCAGAGCCGATCCAGC | Sense |
| 9-R | 9403–9426 | GTAGTCTAAAGCAGAGGCAAATGA | Reverse |
| 10-F | 9216–9237 | GGTGGTAACATACGCCCTGAAT | Sense |
| 10-R | 10977–10998 | CCTAACGGTGGGAGGCTTAGAT | Reverse |

^a^The primers were designed from the Bangkok strain (KJ741266).

**Tab. S1 Comparison on amino acid substitutions among JEV TC4E10_18-9E-Y-T-Cxt-Y-5-11 strain, DH10M978 strain, vaccine strain SA14-14-2, and SA14 strain**

| No. | Protein | Mutation sites | Strain | |  |  |
| --- | --- | --- | --- | --- | --- | --- |
|  |  |  | TC4E10_18-9E-Y-T-Cxt-Y-5-11 | DH10M978 | SA14-14-2 | SA14 |
| 1 | C | 66 | L | L | S | L |
| 2 |  | 70 | R | R | K | K |
| 3 |  | 100 | K | K | R | R |
| 4 |  | 110 | S | S | G | G |
| 5 |  | 120 | I | I | V | V |
| 6 |  | 127 | A | T | A | A |
| 7 | prM | 128 | I | M | M | M |
| 8 |  | 148 | D | D | D | G |
| 9 |  | 184 | A | A | T | T |
| 10 |  | 185 | V | V | M | M |
| 11 |  | 267 | A | A | V | V |
| 12 |  | 276 | S | S | N | N |
| 13 | E | 401 | L | L | F | L |
| 14 |  | 417 | N | N | S | S |
| 15 |  | 423 | M | M | T | T |
| 16 |  | 432 | E | E | K | E |
| 17 |  | 470 | I | I | V | I |
| 18 |  | 471 | T | T | A | T |
| 19 |  | 516 | S | S | A | A |
| 20 |  | 538 | E | E | G | E |
| 21 |  | 558 | Q | Q | H | Q |
| 22 |  | 573 | K | K | M | K |
| 23 |  | 609 | A | A | V | A |
| 24 |  | 621 | T | T | S | S |
| 25 |  | 660 | S | S | A | A |
| 26 |  | 727 | L | V | V | V |
| 27 |  | 733 | K | K | R | K |
| 28 | NS1 | 822 | C | W | W | W |
| 29 |  | 845 | Q | Q | K | K |
| 30 |  | 864 | S | S | A | A |
| 31 |  | 941 | R | R | H | H |
| 32 |  | 969 | N | N | S | S |
| 33 |  | 1000 | L | L | Y | Y |
| 34 |  | 1029 | G | G | D | G |
| 35 |  | 1045 | R | R | K | K |
| 36 |  | 1086 | G | G | S | G |
| 37 |  | 1092 | I | I | V | V |
| 38 |  | 1133 | R | R | M | R |
| 39 |  | 1145 | D | D | H | D |
| 40 |  | 1148 | N | N | K | N |
| 41 |  | 1152 | I | I | V | V |
| 42 |  | 1186 | A | A | V | A |
| 43 | NS2A | 1243 | A | A | T | T |
| 44 |  | 1295 | T | T | S | S |
| 45 |  | 1297 | A | A | T | T |
| 46 |  | 1300 | I | I | V | V |
| 47 |  | 1330 | Q | Q | H | H |
| 48 |  | 1333 | R | R | K | K |
| 49 | NS2B | 1428 | D | D | E | E |
| 50 |  | 1436 | E | E | D | E |
| 51 |  | 1438 | E | E | G | D |
| 52 |  | 1472 | L | L | V | V |
| 53 | NS3 | 1518 | L | L | S | S |
| 54 |  | 1563 | M | M | V | M |
| 55 |  | 1582 | S | S | A | A |
| 56 |  | 1609 | P | P | G | A |
| 57 |  | 1681 | D | D | E | E |
| 58 |  | 1686 | S | S | N | N |
| 59 |  | 1689 | K | K | R | R |
| 60 |  | 1858 | D | D | E | E |
| 61 | NS4A | 2233 | V | V | I | I |
| 62 |  | 2292 | R | R | K | K |
| 63 |  | 2296 | P | P | S | S |
| 64 |  | 2345 | S | S | N | N |
| 65 |  | 2378 | I | I | V | I |
| 66 |  | 2390 | V | V | A | A |
| 67 | NS5 | 2549 | D | D | E | E |
| 68 |  | 2628 | K | K | R | R |
| 69 |  | 2807 | R | R | K | K |
| 70 |  | 2814 | R | R | K | K |
| 71 |  | 2899 | V | V | A | A |
| 72 |  | 2913 | H | H | Y | H |
| 73 |  | 2956 | G | G | D | D |
| 74 |  | 2959 | L | L | R | R |
| 75 |  | 2965 | N | N | D | D |
| 76 |  | 3115 | G | G | E | E |
| 77 |  | 3166 | Q | Q | H | Q |
| 78 |  | 3198 | V | V | A | V |
| 79 |  | 3281 | R | R | K | K |
| 80 |  | 3405 | I | I | V | V |

C: capsid; E: envelope; NS: non-structural; prM: pre-membrane
